# Dynamic Finite Element Model Based on Timoshenko Beam Theory for Simulating High-Speed Nonlinear Helical Springs

**DOI:** 10.3390/s23073737

**Published:** 2023-04-04

**Authors:** Jianwei Zhao, Zewen Gu, Quan Yang, Jian Shao, Xiaonan Hou

**Affiliations:** 1 Institute of Engineering Technology, University of Science and Technology Beijing, Beijing 100083, China; 2 Department of Engineering Mechanics, College of Pipeline and Civil Engineering, China University of Petroleum, Qingdao 266580, China; 3 Department of Engineering, Lancaster University, Engineering Building, Lancaster LA1 4YW, UK

**Keywords:** dynamic finite element analysis, nonlinear helical spring, Timoshenko beam theory, high-speed impacting

## Abstract

Helical springs with nonlinear geometric parameters nowadays have shown great advantages over classical linear springs, especially due to their superior performance in diminishing dynamic responses in high-speed situations. However, existing studies are mostly available for springs with linear properties, and the sole FE spring models using solid elements occupy significant computational resources. This study presents an FE spring model based on Timoshenko beam theory, which allows for high-speed dynamic simulations of nonlinear springs using a beehive valve spring sample. The dynamic results are also compared with the results of the FE model using solid elements and the results of the engine head test and indicate that the proposed FE model can accurately predict dynamic spring forces and the phenomenon of coil clash when simulating the beehive spring at engine speeds of both 5600 and 8000 RPM. The results also indicate that rapid coil impact brings significant spike forces. It should also be noted that the FE spring model using beam elements displays sufficient accuracy in predicting the dynamic responses of nonlinear springs while occupying much fewer computational resources than the FE model using solid elements.

## 1. Introduction

As one of the most fundamental flexible mechanical parts, helical springs are widely applied in various engineering disciplines such as high-speed sports car engines [1], energy-harvesting devices [2] and robotic exoskeletons [3] due to its flexibility and the functionality of storing or absorbing energy. The basic theories for depicting the mechanical properties of helical springs date back to the simple Hook’s law and the relatively more comprehensive Wahl’s spring theory [4], which formulated the relationships between the geometric parameters of a normal helical spring and its static mechanical properties. As for meeting the demand of working in various conditions, helical springs were later designed with irregular shapes, which were found to hardly be studied based on traditional spring theories. Since then, researchers began extending traditional spring theories for including these geometric nonlinearities, for instance, the effects of nonuniform spring ends [5], variable spring pitch [6] and variable coil diameter [7]. These extended theories investigate the influence of the complex internal structures of irregular helical springs on their overall mechanical properties, surpassing the traditional theories that simplify springs as several geometric parameters. Recently, an analytical model was developed to dynamically calculate the dead coils of helical springs during static compressions [8]. 

In addition to static conditions, helical springs are often exposed to dynamic conditions containing high-frequency, rapid-impacting and nonharmonic excitations. Such conditions are usually accompanied by high-value dynamic stresses and therefore result in cracks and the failure of springs. The internal natural vibration of the springs, known as spring surge, was claimed by researchers [9,10,11] as the culprit of the significant increase in dynamic stresses. For modelling the dynamic responses of helical springs, the multibody spring model was proposed by discretizing a helical spring as multiple spring-mass systems [12,13]. However, the accuracy of these models greatly relies on precise estimations of the positions of coil contact, and they can hardly describe the geometric nonlinearities of irregular springs. Based on Wahl’s basic spring theory, the dynamic responses of helical springs can also be assumed as vibrations of a wave [4]. Analytical spring models were developed based on this theory to simulate the spring surge of helical springs working at low-speed situations [14,15]. An improved analytical spring model was also proposed for including the nonlinear parameters and the changing number of active coils, which, however, still failed to predict the significant spring forces caused by coil clash of springs in high-speed situations [8]. Moreover, the finite element method (FEM) was applied to represent the complex geometries of irregular springs working in both low- [16] and high-speed [17] situations. As the considerations of irregular spring shapes and coil contacts during high-speed operations were absent in this research, recent studies have included nearly all the nonlinear factors in a proposed comprehensive FE spring model [1], which can accurately simulate the dynamic spring forces and coil clash in both low- and high-speed situations. Despite superior performance, FE spring models were claimed to occupy significant computational resources, especially, when calculating transient cases. 

For lifting the efficiency of simulating dynamic responses of springs, helical springs were assumed as curved beams based on the Timoshenko [18,19], classical Bernoulli-Euler [20,21,22,23] and refined Bernoulli-Euler [5] beam theories. These methods were mostly used by researchers to investigate the free vibrations of normal helical springs in low-speed situations without considering nonlinear geometries, high-speed dynamic responses and coil clash [24,25,26,27,28]. It was also pointed out that the results of simulating springs with variable pitch and radius by using existing straight beam theories could bring inefficiency and inaccuracy [21]. In this study, an FE model was developed by discretizing spring coils as curved beams based on Timoshenko beam theory to simulate the dynamic responses of irregular helical springs in high-speed working situations (over 5600 RPM engine speeds). Timoshenko beam theory and the developing process of the FE model are also demonstrated. The practical spring sample is a beehive valve spring, which contains nonlinear geometric parameters and shows coil clash phenomenon when used in high-speed car engines. The simulation results of the beehive spring at both 5600 RPM and 8000 RPM engine speeds are compared with the results of the engine head test and the simulation results using the existing FE spring model based on solid elements. The strengths and weaknesses of both of the FE spring models are also determined.

## 2. Timoshenko Beam Theory for Curved Beams

**Figure 1:**
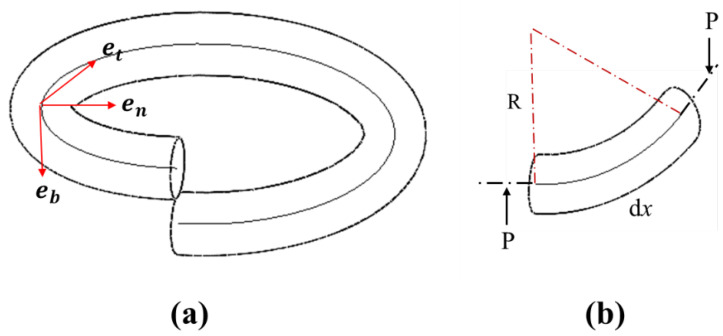
(**a**) One spring coil in the Serret-Frenet coordinates. (**b**) Unit rod element of a spring coil.

Figure 1a displays one entire coil of a normal helical spring. Figure 1b shows the unit rod element of the coil, which is assumed as a curved beam in this study. With this assumption, the helical spring with helical radius *R* is considered to consist of unit beam elements with a circular cross section of radius *r*; $e_{n},\;e_{b}$ and $e_{t}$ denote the normal, binormal and tangent unit vectors in the Serret-Frenet basis [29]; and the curvature κ and torsion τ are given as $\kappa =\cos^{2}\alpha /R$ and τ=sinα·cosα/*R*, respectively.

When the helical spring is subjected to axial compression load *P*, the general governing equations of the curved beam based on Timoshenko beam theory can be expressed based on [30,31,32].

(1) $$\frac{du_{n}}{ds}=\tau u_{b}-\kappa u_{t}+\phi_{b}+\frac{Q_{n}}{GA_{n}}\;,$$

(2) $$\frac{du_{b}}{ds}=-\tau u_{n}-\phi_{n}+\frac{Q_{b}}{GA_{b}}\;,$$

(3) $$\frac{du_{t}}{ds}=\kappa u_{n}+\frac{Q_{t}}{EA_{t}}\;,$$

(4) $$\frac{d\phi_{n}}{ds}=\tau \phi_{b}-\kappa \phi_{t}+\;\frac{M_{n}}{EI_{n}}\;,$$

(5) $$\frac{d\phi_{b}}{ds}=-\tau \phi_{n}+\frac{M_{b}}{EI_{b}}\;,$$

(6) $$\frac{d\phi_{t}}{ds}=\kappa \phi_{n}+\frac{M_{t}}{GI_{t}}$$

(7) $$\frac{dQ_{n}}{ds}=-\rho A_{t}\omega^{2}u_{n}-P\sin\alpha \left(\frac{d\phi_{b}}{ds}+\frac{1}{GA_{n}}\frac{dQ_{n}}{ds}+\tau \phi_{n}-\tau \frac{Q_{b}}{GA_{b}}\right)+P\cos\alpha \left(\frac{d\phi_{t}}{ds}-\kappa \phi_{n}+\kappa \frac{Q_{b}}{GA_{b}}\right)+\tau Q_{b}-\kappa Q_{t}\;,$$

(8) $$\frac{dQ_{b}}{ds}=-\rho A_{t}\omega^{2}u_{b}+P\sin\alpha \left(\frac{d\phi_{n}}{ds}+\frac{1}{GA_{b}}\frac{dQ_{b}}{ds}-\tau \phi_{b}-\tau \frac{Q_{n}}{GA_{n}}+\kappa \phi_{t}\right)-\tau Q_{n}\;,$$

(9) $$\frac{dQ_{t}}{ds}=-\rho A_{t}\omega^{2}u_{t}-P\cos\alpha \left(\frac{d\phi_{n}}{ds}-\frac{1}{GA_{b}}\frac{dQ_{b}}{ds}-\tau \phi_{b}-\tau \frac{Q_{n}}{GA_{n}}+\kappa \phi_{t}\right)+\kappa Q_{n}\;,$$

(10) $$\frac{dM_{n}}{ds}=-\rho I_{n}\omega^{2}\phi_{n}+Q_{b}-PR\cos\alpha \left(\frac{d\phi_{b}}{ds}+\frac{1}{GA_{n}}\frac{dQ_{n}}{ds}+\tau \phi_{n}-\tau \frac{Q_{b}}{GA_{b}}\right)-PR\sin\alpha \left(\frac{d\phi_{t}}{ds}-\kappa \phi_{n}+\kappa \frac{Q_{b}}{GA_{b}}\right)+\tau M_{b}-\kappa M_{t}\;,$$

(11) $$\frac{dM_{b}}{ds}=-\rho I_{b}\omega^{2}\phi_{b}-Q_{n}+PR\cos\alpha \left(\frac{d\phi_{n}}{ds}+\frac{1}{GA_{b}}\frac{dQ_{b}}{ds}-\tau \phi_{b}-\tau \frac{Q_{n}}{GA_{n}}+\kappa \phi_{t}\right)-\tau M_{t}\;,$$

(12) $$\frac{dM_{t}}{ds}=-\rho I_{t}\omega^{2}\phi_{t}+PR\sin\alpha \left(\frac{d\phi_{n}}{ds}-\frac{1}{GA_{b}}\frac{dQ_{b}}{ds}-\tau \phi_{b}-\tau \frac{Q_{n}}{GA_{n}}+\kappa \phi_{t}\right)+\kappa M_{n}\;,$$

where ω and s denote the frequency and the curvilinear coordinate, respectively; ρ,E, G are the material density, the Young’s modulus and the shear modulus, respecitively, when ν is the Poisson coefficient; $\left[u_{n},\;u_{b},u_{t}\right]$, $\left[\phi_{n},\;\phi_{b},\phi_{t}\right]$, $\left[Q_{n},\;Q_{b},\;Q_{t}\right]$ and $\left[M_{n},M_{b},M_{t}\right]$ are the desplacement vector, the rotation vector, the internal force vector and the moment vectors, respectively; and $A_{n},\;A_{b}\;\mathrm{and}\;A_{t}$ represent the area of the circular cross sections of wires, where $A_{n}=A_{b}=\gamma A_{t}=\gamma \pi r^{2}$ and $\gamma =6\left(1+\nu \right)/\left(7+6\nu \right)$ are the Timoshenko shear coefficient.

Timoshenko beam theory therefore relates the forces and moments of the displacements and rotations of the unit rod elements, which allows calculations of the overall spring force according to the applied compression loads. Unlike Euler–Bernoulli beam theory, which neglects shear deformations of the beam, Timoshenko beam theory assumes that the cross section of the beam remains flat during bending [33]. Timshenko beam theory is therefore deemed more suitable to represent the geometry of helical springs that are assumed as a combination of a finite number of short curved beams.

## 3. Dynamic Finite Element Analysis Based on Beam Elements

**Figure 2:**
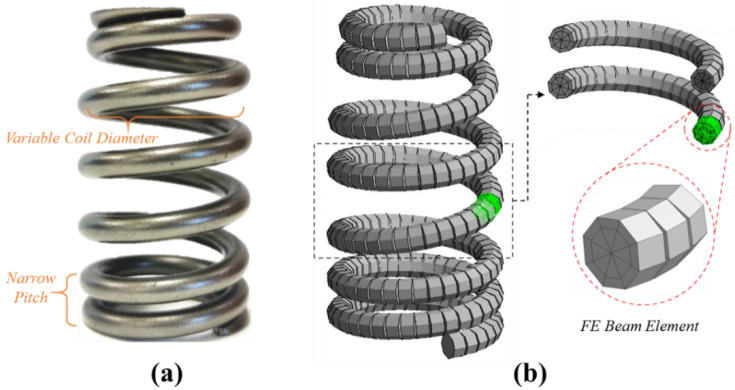
(**a**) The beehive spring sample. (**b**) The FE model of the beehive spring using the Timoshenko beam element.

In this study, a finite element spring model is developed using the commercial software Ansys Workbench, of which beam element is based on Timoshenko beam theory. Figure 2a is the real beehive valve spring product, which is used in high-speed valve train systems of sports car engines. Different from a normal helical spring, the beehive spring contains nonlinear geometric parameters (dead spring ends, narrow spring pitches and variable coil diameters), of which values can be found in Table 1. The geometry model of the beehive spring is developed based on these values, which is then meshed by FE beam elements as shown in Figure 2b. The whole spring coil is split into a finite number of intervals, and each interval is assumed as a short Timoshenko beam element.

**Figure 3:**
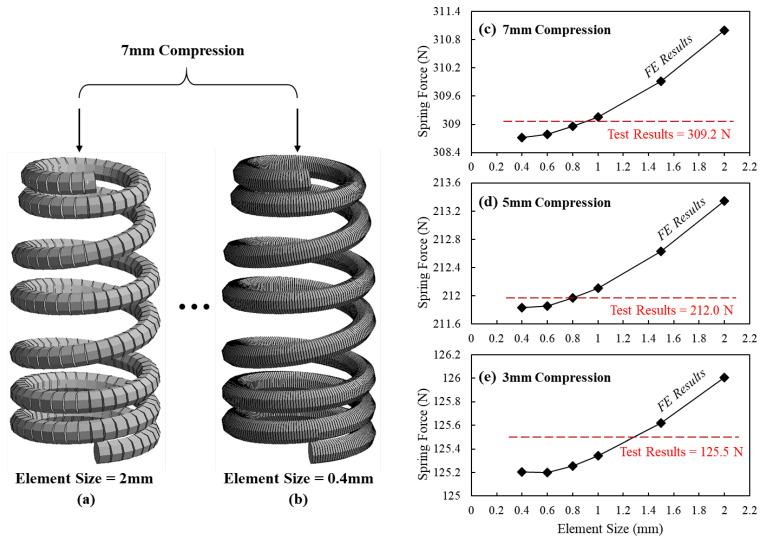
The FE models of beehive spring with (**a**) 2 mm and (**b**) 0.4 mm element sizes and the convergency study of the size of elements at (**c**) 7 mm, (**d**) 5 mm and (**e**) 3 mm compressions.

For determining the number of intervals that are used to split the coil, a convergence study of element sizes is conducted on the FE spring model. First, a 7 mm static compression load is applied on the first coil of the spring from the top end when the last coil is fixed, as they are both dead coils in practice. Second, frictionless contact is defined for the overall spring coil to simulate the coil clash phenomenon. These settings well simulate the practical status of the beehive spring that is installed in the valve train of the sports car engine. Figure 3a,b are the FE spring models meshed by 2 mm and 0.4 mm element sizes, respectively. Correspondingly, the simulated spring forces under 3 mm, 5 mm and 7 mm compression are shown in Figure 3a–c, respectively. By comparison with the results of static spring compression, it is noted that the FE results are converged when the element size is smaller than 1 mm. Hence, the element size of 0.4 mm is selected in this study to ensure accuracy of the FE simulation results.

**Figure 4:**
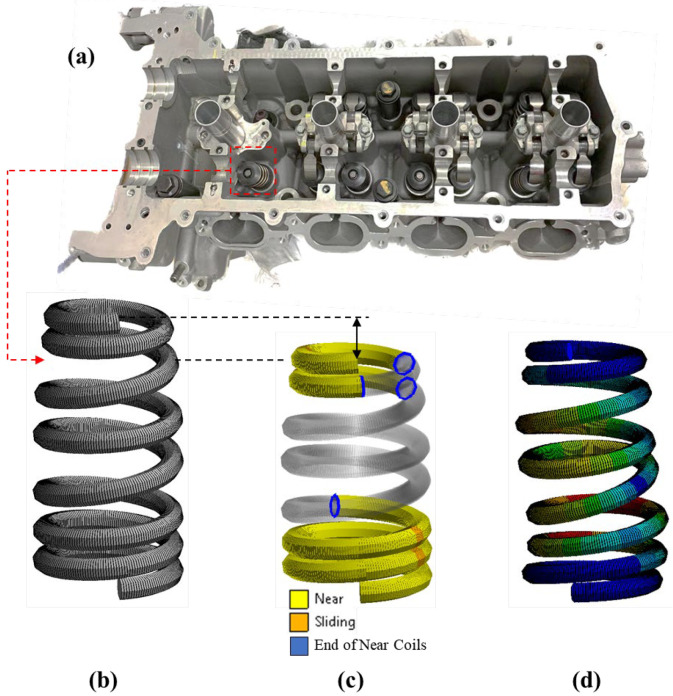
(**a**) The valve train system of a V8 sports car engine. (**b**) The FE spring model with a 0.4 mm element size and its (**c**) static and (**d**) dynamic status.

In practice, the beehive spring is installed in the valve train system with a 7 mm precompression to ensure the closure of valves during high-speed operations. Figure 4a shows the valve train of a V8 sports car engine with the beehive spring installed. The developed FE spring model with a 0.4 mm element size is shown in Figure 4b, and the FE model with a 7 mm precompression is shown in Figure 4c. The yellow and orange colors demonstrate that the coils close to both spring ends have been closed under this compression. When motivated by the overhead cam at high engine speeds, the beehive spring performs nonlinear dynamic responses as shown in Figure 4d. Similar to the static settings, the dynamic loading is exerted on the first coil from the top end of the spring model when the last coil is fixed. As the top spring end is directly actuated by the cam during operations, the cam profile of the valve train is used as the input to the FE simulation. The cam profile of the valve train and the damping ratio are the same as those used in the previous study, where the detailed information of the cam can also be found.

## 4. Results and Discussions

The FE spring model based on Timoshenko beam theory is simulated at both 5600 RPM and 8000 RPM engine speeds. The results of the engine head test at both of the engine speeds are also used to validate the accuracy of the developed FE spring model. In addition, the simulation results of the FE spring model developed in the previous study [1] based on the solid element are compared with the dynamic simulation results obtained in this research. In both of the FE simulations, the spring reaction forces from the bottom end of the spring are recorded to compare with the spring forces obtained from the engine head tests. 

**Figure 5:**
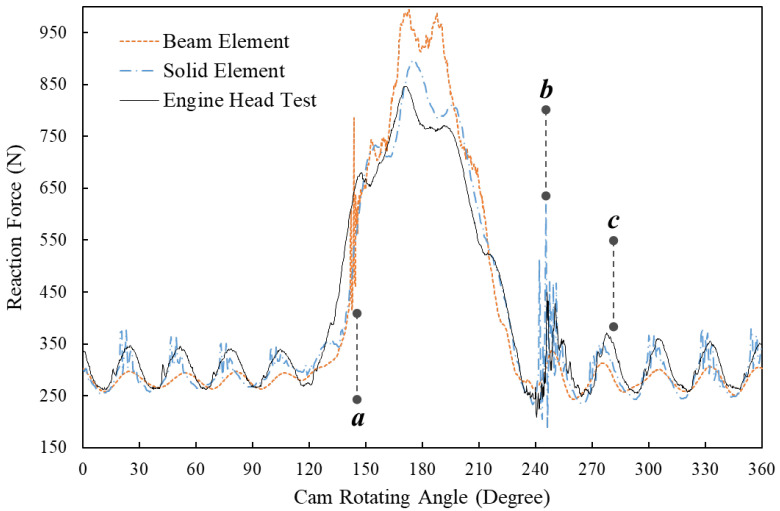
Comparison of the spring reaction forces at 5600 RPM engine speed of the dynamic FE results using beam elements and solid elements and the results of the engine head test.

**Figure 6:**
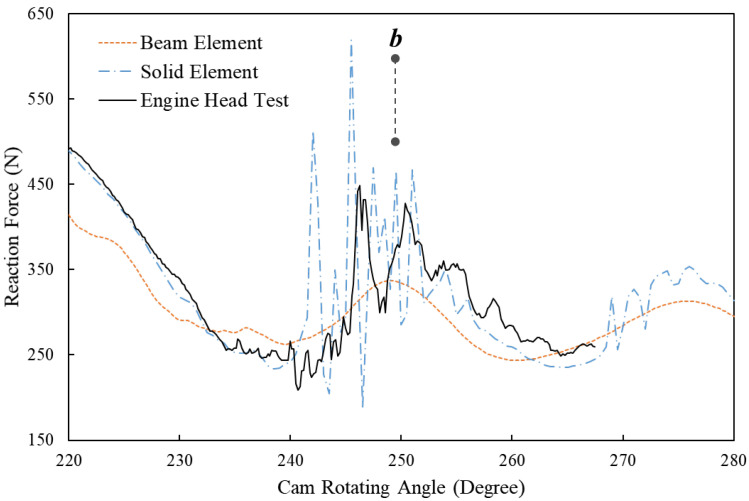
Comparison between the spring reaction forces at 5600 RPM engine speeds of the dynamic FE results using beam elements and solid elements and the results of the engine head test (zoom-in area between the 220-degree and 280-degree cam angles).

Figure 5 compares the FE simulation results that are based on the beam element, the solid element and the results of the engine head test at the engine speed of 5600 RPM. The letters *a*, *b* and *c* in the figure denote three positions when the cam rotates to different angles. At position *a* when the cam angle is around 150 degrees, the simulated reaction force using the beam element shows a rapid increase, which is not observed in the results of the simulation using the solid element and the engine head test. Despite the difference, both of the simulated reaction forces still fit well with the test results after the cam angle of 150 degrees. However, it is noted that the beam element result has a peak force of approximate 960 N at around 170 degrees, while the peak forces of the solid element and the engine test are only around 860 N. At position *b* when the cam angle is around 250 degrees, the FE model using solid element successfully simulates the fluctuating forces at the beginning of the free vibration of the spring, which can also be observed in the results of the engine head test. Figure 6 is the zoom-in area between 220-degree and 280-degree cam angles, which, however, shows that the beam element result fails to predict this fluctuation of reactions force between 240 and 270 degrees. At position *c* and afterwards when the spring is still in the state of free vibration, the FE model of beam elements accurately predicts the vibration frequency of the spring, while its simulated peak values of each vibration wave are around 30 N less than the results for the solid element and the engine head test.

**Figure 7:**
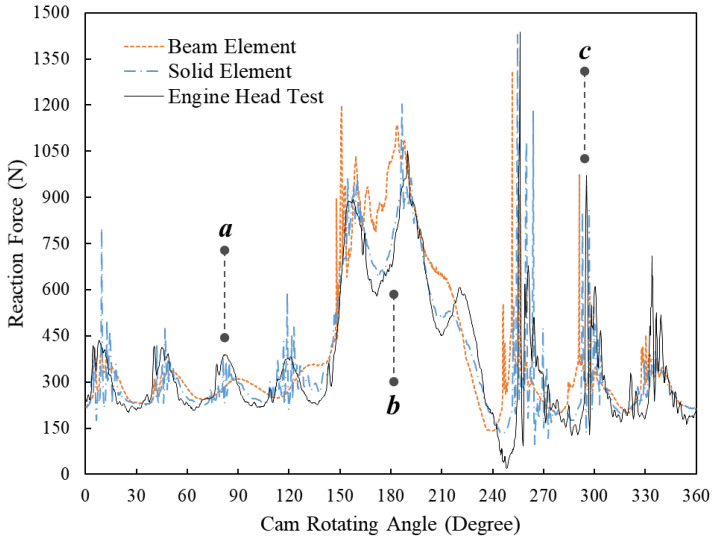
Comparison between the spring reaction forces at 8000 RPM engine speed of the dynamic FE results using beam elements and solid elements and the results of the engine head test.

**Figure 8:**
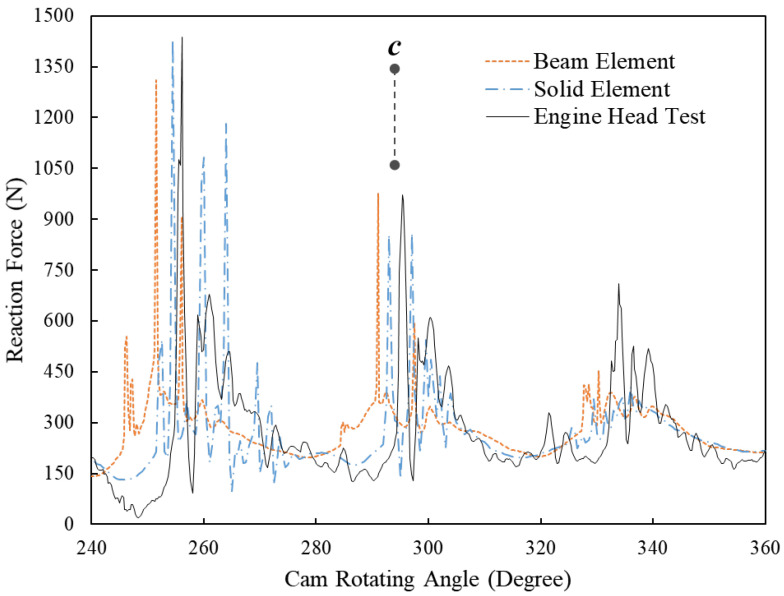
Comparison between the spring reaction forces at 8000 RPM engine speeds of the dynamic FE results using beam elements and solid elements and the results of the engine head test (zoom-in area between the 220-degree and 280-degree cam angles).

Figure 7 displays the dynamic reaction forces of the beehive spring at the 8000 RPM engine speed simulated by FE models using beam elements and solid elements and tested in the engine head. Similar to 5600 RPM engine speed, the FE model using beam elements accurately predicts the frequency of free vibration at around position *a*, while its peak values of each wave are still around 30 N less than those of the other two results. The FE model using beam elements successfully predicts the small fluctuations of reaction force around position *b*, though it fails to simulate the concave point that appeared at around the 180-degree cam angle. The zoom-in area around position *c* is shown in Figure 8, where the significant spring force is proven to have been caused by rapid impacts between spring coils [1]. It is noted that the FE model using beam elements can predict the fluctuations of reaction forces and also the peak values of significant forces at this range as accurately as the FE model using solid elements did.

**Figure 9:**
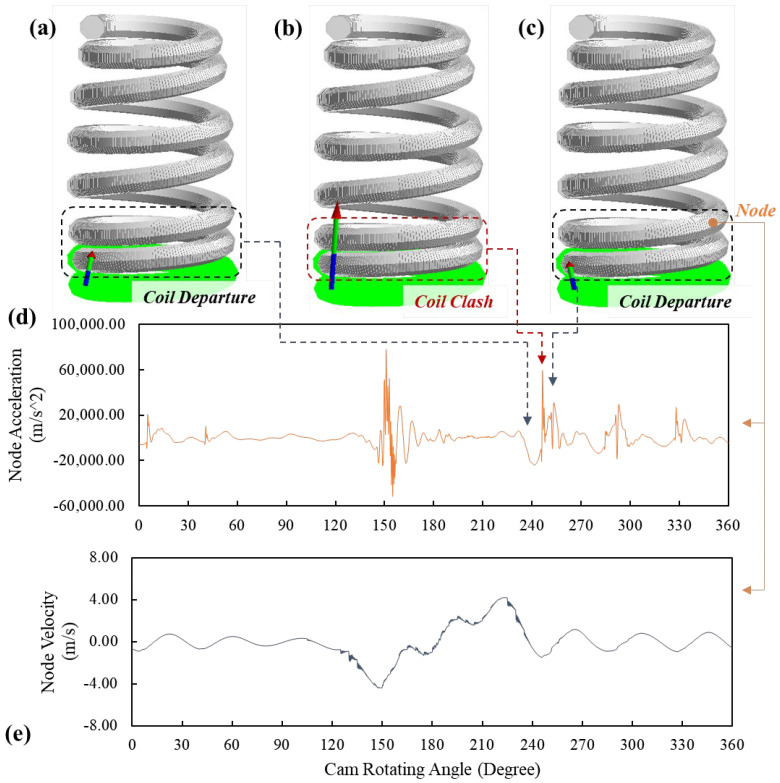
The motion status of the FE spring model at (**a**) 255-degree, (**b**) 256-degree and (**c**) 257-degree cam angles and the (**d**) acceleration and (**e**) velocity of the node on the third coil from the lower spring end.

To explain the significant spring forces, the motion status of the simulated spring model at 255-degree, 256-degree and 257-degree cam angles are shown in Figure 9a–c, respectively. The velocity and acceleration of an element node on the third coil from the lower spring end are extracted and shown in Figure 9d,e, respectively. At 255-degree cam angle, the third coil separates from the second coil (Figure 9a) when it is moving toward the second coil, as shown in Figure 9e. Next, the third coil impacts the second coil, which causes the so-called coil clash phenomenon. The significant increase of node acceleration at the 256-degree cam angle (shown in Figure 9b) also indicates the impact, and shortly the third coil moves away from the second coil, as shown in Figure 9e. As the engine speed is 8000 RPM, the time period of the entire cam rotation (360 degrees) is 0.015 s. Therefore, the process of the impact at the 256-degree cam angle between the second and third coils is completed in less than 2.1 × 10^−4^ s. This also shows that the significant spring forces generated at the 256-degree cam angle in Figure 7 and Figure 8 are caused by the coil clash between the second and third coils. These findings also coincide with the conclusions drawn from the previous research [1] that developed an FE spring model using solid elements to simulate the dynamic responses of helical springs at high engine speeds.

## 5. Conclusions

Based on Timoshenko beam theory, an FE model of the beehive spring using beam elements was developed in this study to simulate its high-speed dynamic responses. Different from the existing spring models based on beam theories, the developed FE model includes the effect of nonlinear geometric parameters of the beehive spring and rapid impacts between spring coils. A convergence study was conducted on the developed model to determine the size of beam elements for accuracy.

A comparative study was also conducted between the results of the dynamic simulations of the FE spring models using beam elements and solid elements and the results of the engine head tests. The FE simulations were run at both 5600 RPM and 8000 RPM engine speeds. It was found that the FE model using beam elements is able to simulate the overall dynamic spring forces, though the FE model using solid elements still shows better accuracy in predicting the peak forces at 5600 RPM. Nonetheless, the FE model using beam elements accurately simulates the dynamic vibrations, the peak forces, and the significant forces. In addition, the FE model using beam elements accurately predicts the significant spike forces generated during the stage of free vibrations. Extracting the acceleration and velocity results of the node on the spring coil of the FE model using beam elements reveals that the significant spike forces are caused by rapid impacts between adjacent coils, which coincides with the conclusion drawn from the studies of the FE model using solid elements.

## Figures and Tables

**Table 1:** The geometric properties of the beehive spring sample.

Coil Revolution	Helix Height (mm)	Spring Pitch (mm)	Coil Diameter (mm)
1	3.865	5.73	22.25
2	9.132	4.804	22.25
3	17.77	12.47	22.25
4	26.57	5.134	22.25
5	35.21	12.14	21.405
6	43.03	3.484	10.017
7	47.35	5.152	18.35

## Data Availability

No new data were created in this study.
